# Activation of an exonic splice‐donor site in exon 30 of *CDK5RAP2* in a patient with severe microcephaly and pigmentary abnormalities

**DOI:** 10.1002/ccr3.663

**Published:** 2016-08-23

**Authors:** Alistair T. Pagnamenta, Malcolm F. Howard, Samantha J. L. Knight, David A. Keays, Gerardine Quaghebeur, Jenny C. Taylor, Usha Kini

**Affiliations:** ^1^ National Institute for Health Research Biomedical Research CentreWellcome Trust Centre for Human GeneticsUniversity of OxfordOxfordUK; ^2^ Institute of Molecular PathologyViennaAustria; ^3^ Department of NeuroradiologyOxford University Hospitals NHS Foundation TrustOxfordUK; ^4^ Department of Clinical GeneticsOxford University Hospitals NHS Foundation TrustOxfordUK

**Keywords:** *CDK5RAP2*, exome, exonic splice‐donor, microcephaly, pigmentation abnormalities

## Abstract

This report constitutes the first report of a cryptic exonic splice‐donor site in *CDK5RAP2*, highlights the importance of evaluating novel splice mutations, and suggests that the phenotypic range associated with *CDK5RAP2* mutations may include skin pigmentary abnormalities.

## Introduction

*CDK5RAP2* is one of the less commonly reported genes for autosomal recessive primary microcephaly (MCPH). Although it was identified as the gene underlying MCPH3 (OMIM#604804) over a decade ago 1, only nine families are reported in the literature. Until recently, the only patients described were from consanguineous families (Table S1). A 2015 study has shown that mutations in this gene may also result in a mild form of Seckel syndrome 2. While the majority of reported mutations represent loss‐of‐function (LoF) alleles, another recent study suggested that milder missense mutations may result in structural defects limited to the corpus callosum 3. To test the theory that there is a genotype–phenotype correlation, it is important to describe additional patients (MCPH and non‐MCPH phenotypes) with biallelic *CDK5RAP2* mutations and evaluate novel mutations to determine whether there is any residual function. This will be especially important for splice mutations where the consequence of the mutation at the RNA level is hard to predict.

**Figure 1:**
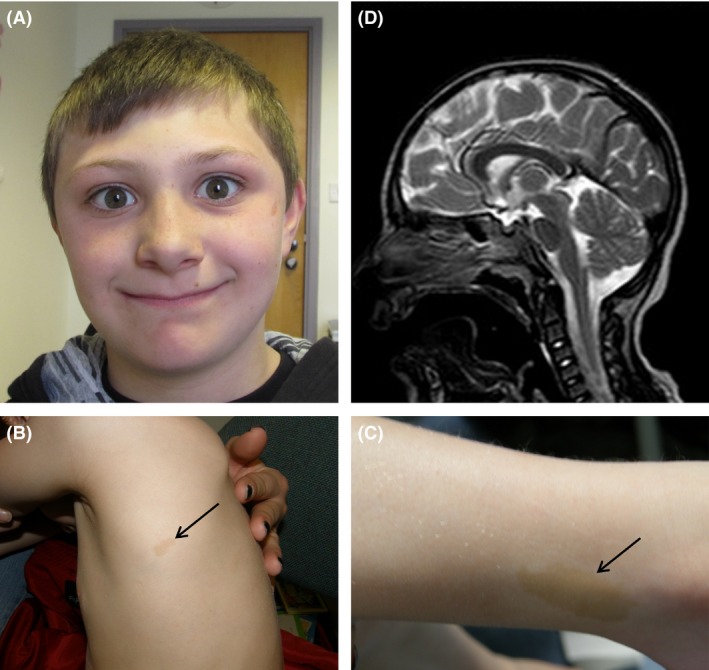
Clinical images showing microcephaly and pigmentation anomalies. (A) Photograph showing the small head size (−5 to −6 SD), sloping forehead, and prominent nose. (B, C) Arrows indicate the multiple café au lait patches. (D) MRI brain scan (sagittal view) showing a small brain with normal corpus callosum.

Here, we describe BRC081, a severely microcephalic boy (OFC −5.5 SD) with moderate learning difficulties, severe behavioral problems, and multiple *café au lait* macules >0.5 cm in diameter on his skin (Fig. 1A–C and Supporting information). He attends a special needs school and has been assessed by an educational psychologist (using the Wechsler Intelligence Scale for children) to be functioning at the mental age of 5 years at a chronological age of 11 years. He has a statement of special educational needs. The patient's hearing was tested by an audiologist and reported to be normal. MRI scans showed no migrational/callosal abnormalities (Fig. 1D). Parent–parent–child trio whole‐exome sequencing (WES) was performed at the WTCHG in Oxford using SeqCap EZ Human Exome Library (NimbleGen) and the HiSeq2000 (Illumina), yielding a mean target coverage of 50–77x (Table S2). No high‐confidence *de novo* mutations were detected. Focussing on an autosomal recessive model, the only plausible variants identified were compound heterozygous mutations in *CDK5RAP2* (NM_018249.5)*:* a c.4604+1G>C transversion in the splice‐donor site of exon 30 and a c.3097delG frameshift in exon 23 (p.V1033 fs*41). Sanger sequencing confirmed that c.4604+1G>C was maternally inherited while c.3097delG was paternal.

Some *CDK5RAP2* mutations described previously have been found to be recurrent (Table S1). For instance, c.246T>A;p.Y82* (identified in two Pakistani kindreds) is listed in dbSNP142, while c.4441C>T;p.R1481* (identified in three recent studies) is present at an allele frequency of 0.0057% across 60,706 WES datasets from ExAC. In contrast, neither of the mutations found in BRC081 are in ExAC and to our knowledge have not previously been identified in microcephalic individuals.

**Figure 2:**
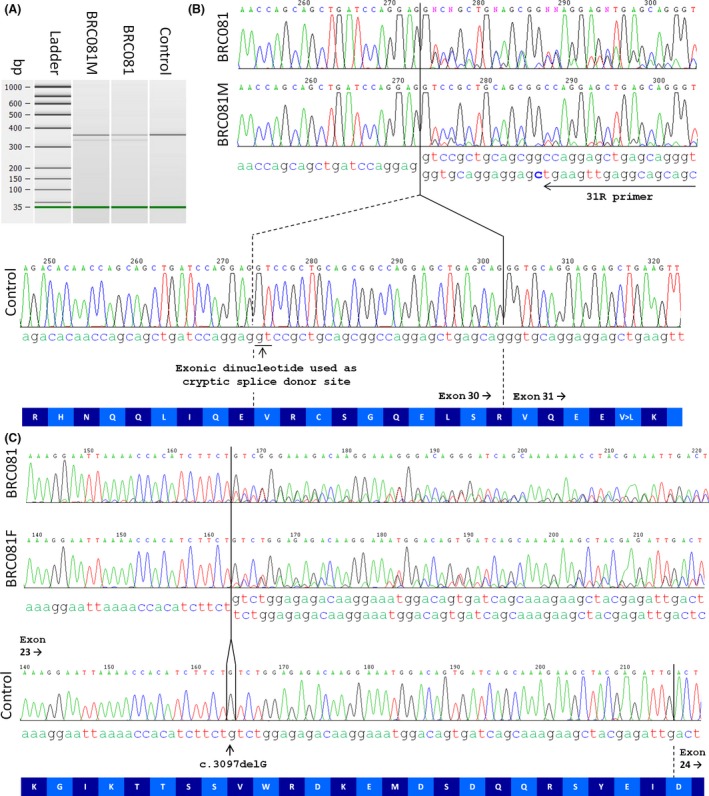
Analysis of the *CDK5RAP2* mutations at the RNA level. (A) Bioanalyzer image showing a 371‐bp product as expected for the exon 28–31 RT‐PCR product. A lower band was also observed for BRC081 and his mother, consistent with the 342‐bp product predicted by MaxEntScan algorithm. A similar pattern was also seen using the smaller exon 29–31 RT‐PCR product (data not shown). Relative quantification of RT‐PCR products is shown in Table S3. (B) Sanger sequencing of the exon 28–31 RT‐PCR product confirmed the use of a cryptic splice‐donor site in the patient, 29 bp upstream of the usual splice site. The sequence for the frameshifted transcript is weaker in the mother than it is for BRC081 (where both chromosomes carry LoF mutations). This observation is consistent with the relative band intensities seen in panel (A). The position of the 31R primer is shown with an arrow. (C) Sanger sequencing of the exon 22–24 RT‐PCR product confirms that the c.3097delG transcript is also expressed. Again, the sequence for the frameshifted transcript is slightly weaker in the father than it is for BRC081 (where both chromosomes carry LoF mutations).

We obtained mRNA obtained directly from leukocytes and by RT‐PCR replicated the finding that exon 32 is alternatively spliced 4. Therefore, to assess the effect of c.4604+1G>C, we used a reverse primer positioned in exon 31. A lower band was observed for samples from BRC081 and his mother that was not seen in the control (Fig. 2A). Sanger sequencing confirmed the usage of a cryptic exonic splice‐donor site 29 bp upstream, consistent with the in silico prediction (the cryptic spice site giving a maximum entropy model score of 2.95 compared to 6.13 and −2.15 for the wild‐type and mutated splice‐donor site, respectively) which would result in p.V1526 fs*15 at the protein level (Fig. 2B). The GT dinucleotide used as the cryptic splice‐donor site also overlaps a predicted exonic splice enhancer site (Fig. S1). Of the splice mutations previously identified in *CDK5RAP2*, c.4005‐15A>G and c.4005‐9A>G both create superior intronic splice sites 1, 2, while c.383+1G>C and c.4005‐1G>A were not evaluated at the RNA level 2, 5. This report constitutes the first report of a cryptic exonic splice‐donor site in *CDK5RAP2*.

Transcripts harboring frameshift mutations are often subject to NMD 6. In the patient, both the frameshift and the splice mutations are effectively nonsense alleles and so one might expect both to undergo NMD. In contrast, the mother harbors a wild‐type copy of *CDK5RAP2 in trans* with the splice mutation and only the latter would be expected to lead to NMD. We speculate that this may explain the reason why the relative intensity of the bands/sequence trace corresponding to the aberrantly spliced RNA was weaker in the maternal sample than for BRC081 (Fig. 2A and B, Table S3).

In situations where aberrant splicing is detected but the reading frame is maintained, one might predict a milder phenotype (i.e., isolated agenesis of the corpus callosum). Exons 19–21 (NM_001272039.1) and 32 (NM_001011649.2) 4 of *CDK5RAP2* are known to be alternatively spliced, and therefore, any nonsense mutations involving these exons should also be interpreted with caution. For BRC081, RNA analysis helped confirm that both mutations lead to frameshifts involving canonical exons and so we can be confident in our assertion that they are likely to result in LoF.

Comparison of BRC081 with published MCPH3 cases (Table S1) shows that severe microcephaly is the predominant diagnosis. Historically, there may have been a bias in terms of which patients have been selected for *CDK5RAP2* analysis (e.g., linkage to MCPH3 locus and/or Sanger analysis) in the years after the initial disease association was reported 1. But now exome sequencing is relatively commonplace, this large gene is being routinely tested in a much greater variety of patients. Nevertheless, there could still be biases in terms of how filtering is performed and results reported. Although the range of growth restriction has been discussed previously 7, other variable features co‐occur. Structural abnormalities of the brain included simplified gyral pattern in three patients, agenesis/hypogenesis of the corpus callosum in four, and holoprosencephaly, lissencephaly, pachygyria plus an interhemispheric cyst in one. Mild–moderate developmental delay was reported in all affected individuals, and no patients were reported to have severe or profound intellectual disability. Global developmental delay was not always seen as some skill areas were spared. Head size did not correlate with the degree of intellectual disability; for instance, the Lancaster et al. case had only mild–moderate developmental delay despite an OFC of −13.2 SD. Hearing loss has been reported in four patients, while behavioral problems such as hyperactivity, aggression, temper tantrums, and socially inappropriate immature behavior have now been reported in three. Pigmentary anomalies have been noted but were not reported to be a significant finding in *CDK5RAP2* patients. Including BRC081 (Fig. 1B and C) and the three Seckel syndrome cases 2, seven patients are now described with skin pigmentary abnormalities, which range from hypopigmentation to hyperpigmentation (Table S1).

*CDK5RAP2* encodes a centrosomal protein that is important in spindle formation and cellular proliferation and interacts with pericentrin (a protein with several coiled‐coil domains encoded by *PCNT*) 8. It is interesting to note that several patients with microcephalic osteodysplastic primordial dwarfism type 2 (OMIM#210720), a related centrosome‐based microcephaly disorder caused by mutations in *PCNT*, have also been reported to have anomalies of skin pigmentation 9, 10.

In conclusion, our study suggests that the phenotypic range associated with *CDK5RAP2* mutations may include behavioral and pigmentary abnormalities and the exonic splice‐donor site we identified highlights the importance of assessing novel splice mutations for genes such as *CDK5RAP2* where mutation severity may impact on the phenotypic presentation.

## Conflict of Interest

The authors declare no conflict of interest.

## Supporting information


**Appendix S1.** Methods
**Figure S1.** Results of ESEfinder for exon 30 of *CDK5RAP2*.
**Table S1.** Comparison of the genotypic and phenotypic details of patients reported with *CDK5RAP2* mutations.
**Table S2.** Target region coverage statistics.
**Table S3.** Quantification of RT‐PCR products using the 2100 Bioanalyzer.
